# Ovarian hyperstimulation revealing a functional gonadotroph adenoma: a case report

**DOI:** 10.11604/pamj.2025.51.57.47982

**Published:** 2025-06-25

**Authors:** Amel Rahal, Toufik Bennafaa, Hanane Kherrab, Nassima Djennane, Malha Azzouz

**Affiliations:** 1 Department of Endocrinology, Bologhine Hospital, Algiers, Algeria,; 2 Department of Neurosurgery, Mohamed Lamine Debaghine Hospital, Algiers, Algeria,; 3 Department of Anatomopathology, Mohamed Lamine Debaghine Hospital, Algiers, Algeria

**Keywords:** Infertility, hyperestrogenism, stimulated ovaries, pituitary macroadenoma, case report

## Abstract

We report the case of a functional gonadotropic adenoma (FGA) in a 32-year-old woman, initially revealed by ovarian hyperstimulation. Hormonal investigations strongly suggested an FGA; pelvic ultrasound revealed macrofollicular ovarian enlargement. Despite a clear clinical picture, diagnosis was delayed until the appearance of cranial tumor syndrome with ophthalmological impairment and pituitary dysfunction. The ophthalmological emergency necessitated urgent surgery, allowing both decompression of the optic pathways and a reduction in hyperestrogenism. Despite their rarity, it is essential to recognize FGA to avoid potentially fatal complications.

## Introduction

Functional gonadotroph pituitary adenomas are rare, representing 2.9% to 8.1% of gonadotroph and non-functioning pituitary adenomas [1]. Clinical presentation varies with age and sex: more pronounced in young and premenopausal women, and often subtle in postmenopausal women and men. Despite characteristic features, a lack of awareness may delay diagnosis, increasing the risk of serious complications. We present a case of FGA in a reproductive-age woman, with a review of the literature.

## Patient and observation

**Patient information:** a 32-year-old married, nulliparous woman was referred to our endocrinology department for assessment of cranial tumor syndrome. She had a prior history of emergency exploratory laparotomy for suspected ovarian torsion and was being followed in gynecology for couple infertility.

**Clinical findings:** the patient presented with severe headaches, visual disturbances (reduced visual acuity, visual field defects), menstrual irregularities, and severe asthenia.

**Figure 1:**
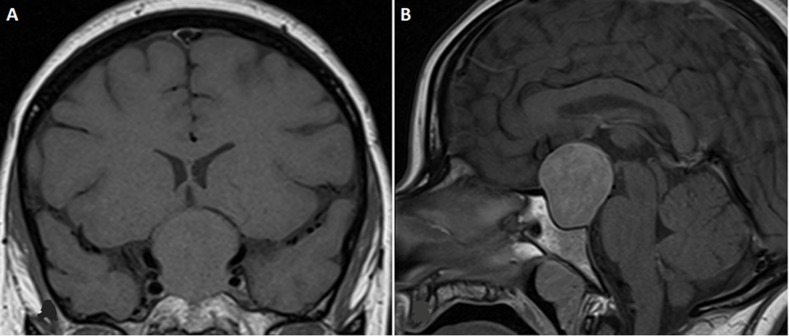
A) pituitary macroadenoma: iso-intense on coronal T1; (B) with moderate and heterogeneous gadolinium enhancement on sagittal T1 sequences

**Figure 2:**
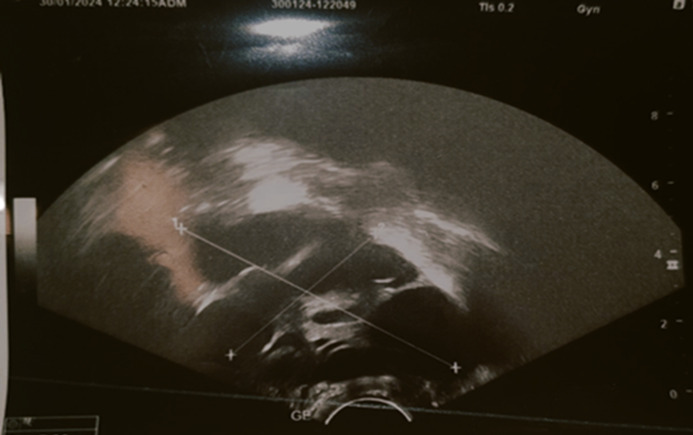
ultrasound appearance of enlarged, macrofollicular stimulated ovaries

**Diagnostic assessment:** clinical suspicion was raised due to cycle irregularities and a history of ovarian torsion. Hormonal investigations showed tumor-level estradiol >6000 pg/mL, elevated follicle-stimulating hormone (FSH): 30 mIU/mL, normal luteinizing hormone (LH): 3.23 mIU/mL, consistent with FGA (Table 1). Prolactin was markedly elevated (639 ng/mL), suggesting possible mixed adenoma, but further assessment revealed isolated thyrotropic deficiency. The presence of cranial tumor syndrome requires pituitary MRI, which reveals a pituitary macroadenoma measuring 36 x 30 mm in diameter, elevating the optic chiasm and the floor of the third ventricle without cavernous sinus invasion or hydrocephalus (Figure 1). Ophthalmological exam confirmed visual acuity reduction (2/10 in the right eye and 5/10 in the left eye), bilateral papillary pallor, and bitemporal hemianopia on visual field testing. In front of the cycle disorders, a pelvic ultrasound revealed large ovaries measuring 10 cm on the right and 12 cm on the left with macrocysts (Figure 2).

**Therapeutic interventions:** given the severity of the ophthalmologic syndrome and the clinical presentation of ovarian hyperstimulation, the patient underwent urgent endoscopic endonasal transsphenoidal surgery. Preoperatively, she received cabergoline (1 mg/week), two injections of somatuline (120 mg).

**Figure 3:**
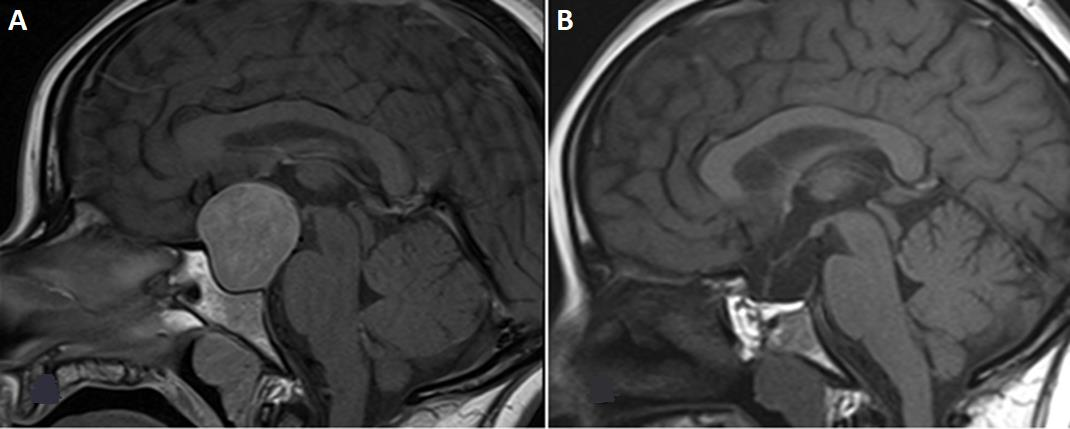
A) sagittal T1-weighted images preoperatively; (B) and postoperatively demonstrate the absence of residual tumor; sagittal T1-weighted images postoperatively reveal optic tract decompression, with the posterior pituitary bright spot clearly visible

**Follow-up and outcome of interventions:** postoperative assessment showed normalization of prolactin and an undetectable level of oestrogen. Complete anterior pituitary insufficiency (corticotropic, thyrotropic, and gonadotropic axes) and central diabetes insipidus, managed medically. Follow-up magnetic resonance imaging (MRI) (3 months) showed no residual tumor and optic chiasm decompression. The posterior pituitary bright spot was not visible (Figure 3).

**Figure 4:**
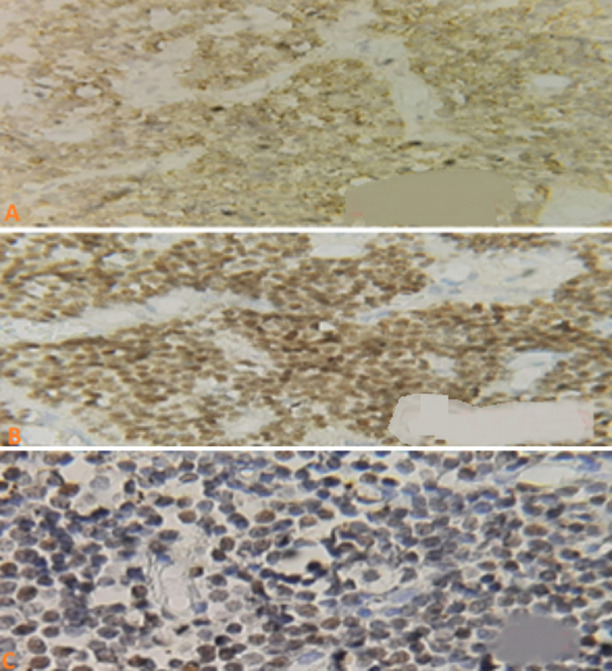
immunohistochemical study: (A,B,C) FSH+GATA3+ SF1- positive immunostaining for SF1 and GATA 3, suggestive of a gonadotropic adenoma

**Histopathological findings:** microscopy showed a monomorphic endocrine tumor with cords and spans of medium-sized cells with weakly eosinophilic cytoplasm. Immunohistochemistry was negative for adrenocorticotropic hormone (ACTH), LH, FSH, thyroid-stimulating hormone (TSH), growth hormone (GH), and prolactin, but diffusely positive for GATA3 and SF1 (over 60% nuclear expression). Ki-67 index: 1% (Figure 4).

**Patient perspective:** the patient reported significant improvement in headaches and visual acuity. She wishes to undergo fertility-inducing therapy, considering her advancing age.

**Informed consent:** the patient has signed a consent form for the publication and use of these data.

**Ethics approval and consent to participate:** the patient has signed a consent form for the publication and use of these data. We have the agreement of the committee of ethics (CEB 00 2025 / 01) of our public hospital for the publication of our case.

## Discussion

### Clinical presentation at diagnosis

**In women:** the clinical presentation of FGAs varies: symptomatic in children and premenopausal women, but more subtle in postmenopausal women. In premenopausal women, FGAs typically present with: menstrual disorders (oligomenorrhea, amenorrhea, menorrhagia), infertility [1,2]. Rarely, ovarian hyperstimulation syndrome (OHSS), with risks of cyst rupture or ovarian torsion [3]. In postmenopausal women, clinical signs resemble those of non-functioning macroadenomas, as the ovaries no longer respond to FSH stimulation. Elevated gonadotropins due to menopause may mask the diagnosis, but a divergence between FSH and LH levels could suggest a gonadotroph adenoma [4]. In our patient, the diagnosis could have been suspected earlier during her first laparotomy for presumed ovarian torsion, given the findings of enlarged, multicystic ovaries.

The stimulated ovaries, often macrofollicular (unlike polycystic ovary syndrome, where follicles are small), alerted the clinician [1]. In our patient, the ovaries measured 12 cm (left) and 10 cm (right), with macrofollicles ranging from 25-35 mm (right) and 22-32 mm (left) (Figure 2).

**In men:** FGA may cause testicular hypertrophy or erythrocytosis [4,5]. In children, they may present as precocious puberty [3].

### Diagnostic laboratory findings

**In women:** laboratory workup is crucial, often revealing hyperestrogenism with paradoxically elevated FSH and normal LH levels [4]. Prolactin levels may be elevated, usually due to stalk effect or hyperoestrogenism.

**In men:** hormonal profiles vary, with elevated or normal FSH, and normal, low, or high LH and testosterone [6]. In our patient, hormonal findings were characteristic: tumor-level estradiol (>3000 pg/mL), very high FSH (29 mIU/mL), and normal LH (Table 1). Prolactin was markedly elevated (600 ng/mL), suggesting a mixed adenoma, but immunohistochemistry ruled this out. Compared to other series, prolactin levels in FGAs rarely exceed 500 ng/mL. In our patient, hyperprolactinemia likely resulted from tumor-induced hyperestrogenism and significant pituitary stalk compression.

**Table 1:** hormonal profile before and after surgery

Hormone	Preoperative	Postoperative	Normal range
Estradiol (pg/mL)	6000	<5	12.5 - 165
FSH (mIU/mL)	30.31	1.86	3.31 - 12
LH (mIU/mL)	5.19	0.72	2.27 - 13.52
Prolactin (ng/mL)	639	0.5	5 - 35
Cortisol nmol/l	329.76	100	101 - 537
FT4 pmol/l	6.83	17	12 - 22

FSH: follicle-stimulating hormone; LH: luteinizing hormone

**Pituitary imaging:** most FGAs are macroadenomas; microadenomas are rarer [3]. MRI is essential for surgical planning. Our patient's MRI showed typical features of a macroadenoma without invasion.

### Therapeutic management

**Objectives:** reduce hormone secretion to prevent OHSS; decompress optic pathways.

### Treatment options

**Medical therapy:** dopamine agonists (e.g. bromocriptine, cabergoline): restore ovulation, reduce tumor in ~44% [7]. Somatostatin Analogs may have anti-secretory and anti-proliferative effects. Oral contraceptives: temporary suppression, but risk of escape [1]. GnRH agonists may worsen tumor growth [3]. In our case, surgery was prioritized due to visual compromise.

**Surgical therapy:** transsphenoidal surgical resection is considered the gold standard treatment for functioning gonadotroph adenomas. This approach enables both tumor removal, restoration of physiological gonadotropin secretion, and improvement of associated symptoms such as menstrual irregularities, ovarian hyperstimulation syndrome (OHSS), and ovarian cystic enlargement [3,4,7]. As demonstrated by several cases described in the literature, as well as our own observation, ovarian hypersecretion (OHS) can progress to a gynecological surgical emergency and threaten the patient's life [3].

**Anatomopathological study:** in our case, the initial anatomopathological study showed an immunonegative non-secreting adenoma requiring immunohistochemistry with complementary transcription factors. Immunohistochemical evaluation of pituitary transcription factors revealed positive immunostaining for SF1 and GATA 3, suggestive of a gonadotropic adenoma, concordant with our clinical and biological data (Figure 4).

**Radiotherapy:** reserved for residual or recurrent tumors [4]. Currently, control imaging is reassuring in our patient.

**Postoperative follow-up:** imaging showed no residual tumor, and hormonal workup confirmed surgical success (undetectable estradiol). Pelvic ultrasound revealed ovarian volume reduction and cyst resolution. The patient, now 33 and childless, desires pregnancy induction. Current gonadotroph deficiency makes spontaneous pregnancy unlikely, necessitating gonadotropin stimulation. Similar cases have achieved successful pregnancies with hormonal induction [8]. Given the high recurrence risk, rigorous clinical, hormonal, and imaging surveillance is essential during and after induction therapy.

## Conclusion

Functional gonadotropic adenomas, though rare, can have dramatic and life-threatening presentations, especially in women of reproductive age. Early recognition based on clinical, hormonal, and imaging criteria is essential. Surgery remains the cornerstone of treatment. Long-term monitoring is crucial due to the risk of recurrence.
